# Data reduction for X-ray serial crystallography using machine learning

**DOI:** 10.1107/S1600576722011748

**Published:** 2023-02-01

**Authors:** Vahid Rahmani, Shah Nawaz, David Pennicard, Shabarish Pala Ramakantha Setty, Heinz Graafsma

**Affiliations:** a Deutsches Elektronen-Synchrotron DESY, Notkestraße 85, 22607 Hamburg, Germany; b Mid-Sweden University, Sundsvall, Sweden; SLAC National Accelerator Laboratory, Menlo Park, USA

**Keywords:** serial crystallography, data reduction, machine learning, feature extraction

## Abstract

Serial crystallography experiments produce massive amounts of experimental data. Yet in spite of these large-scale data sets, only a small percentage of the data are useful for downstream analysis. Thus, it is essential to differentiate reliably between acceptable data (hits) and unacceptable data (misses). To this end, a novel pipeline is proposed to categorize the data, which extracts features from the images, summarizes these features with the ‘bag of visual words’ method and then classifies the images using machine learning. In addition, a novel study of various feature extractors and machine learning classifiers is presented, with the aim of finding the best feature extractor and machine learning classifier for serial crystallography data. The study reveals that the oriented FAST and rotated BRIEF (ORB) feature extractor with a multilayer perceptron classifier gives the best results. Finally, the ORB feature extractor with multilayer perceptron is evaluated on various data sets including both synthetic and experimental data, demonstrating superior performance compared with other feature extractors and classifiers.

## Abstract 2

A machine learning method for distinguishing good and bad images in serial crystallography is presented. To reduce the computational cost, this uses the oriented FAST and rotated BRIEF feature extraction method from computer vision to detect image features, followed by a multilayer perceptron (neural network) to classify the images.

## 1. Introduction

In recent years, serial femtosecond crystallography (SFX) has made remarkable progress in the measurement of macromolecular structures and dynamics using intense femtosecond-duration pulses from X-ray free-electron lasers (FELs) (Wiedorn *et al.*, 2018 ▸; Chapman *et al.*, 2011 ▸). It is well understood that intense X-ray pulses can produce strong diffraction patterns from weakly diffracting crystals. However, the process relies on an effect known as ‘diffraction before destruction’, where the crystal is completely destroyed by the FEL pulse. Therefore, the experiment requires patterns from many crystals, resulting in large quantities of data. For example, SFX experiments at the Linac Coherent Light Source (LCLS) produce images at a rate of 120 Hz, producing data volumes usually amounting to tens of terabytes. Yet despite these large-scale data sets, only a small percentage of the data may actually contain diffraction patterns from the target of interest.

For example, a typical SFX experiment at the European X-ray FEL involving lysozyme produced 749 874 images in 83 min of measurement time at 150 pulses per second, of which 25 193 images (3.4%) were observed to contain diffraction from a protein crystal, as identified in the offline analysis (Wiedorn *et al.*, 2018 ▸). In addition, new free-electron laser facilities such as LCLS-II will handle experiments with repetition rates over 100 kHz, resulting even higher data volumes (Galayda, 2018 ▸). These scenarios present an imperative challenge for the efficient processing and analysis of diffraction data obtained during SFX experiments.

**Figure 1:**
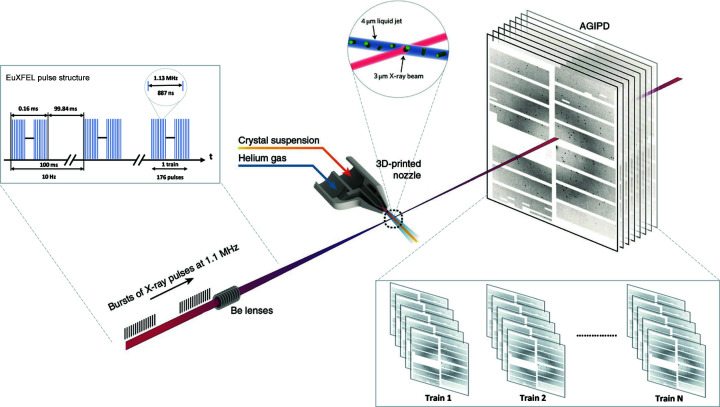
A typical SFX experiment at the European XFEL, producing bursts of X-ray pulses at a megahertz repetition rate, repeating at 10 Hz frequency. Note that the diffraction from the sample is measured using an adaptive gain integrating pixel detector (AGIPD), which is capable of measuring up to 3520 pulses per second at megahertz frame rates (Wiedorn *et al.*, 2018 ▸).

In a typical SFX experiment (Fig. 1 ▸), diffraction patterns may register single-crystal ‘hits’ as well as empty shots (‘misses’) and multiple-crystal hits. In other words, diffraction patterns may register Bragg peaks from crystal hits that are otherwise absent in empty shots. Considering the nature of SFX experiments, it is clear that only crystal hits with Bragg peaks are useful for downstream analysis (Wiedorn *et al.*, 2018 ▸). Therefore, existing statistical methods utilize peak finding to identify and discern diffraction patterns that contain Bragg peaks and remove any patterns which only contain empty shots, resulting in considerable data reduction (Hadian-Jazi *et al.*, 2021 ▸, 2017 ▸; Barty *et al.*, 2014 ▸; Mariani *et al.*, 2016 ▸; Thayer *et al.*, 2017 ▸). Typically, peak finding methods attempt to find all the Bragg peaks in these diffraction patterns, which can be computationally expensive. In addition, existing methods require carefully crafted parameters from human experts in the field.

More recently, the astonishing success of image classification with machine learning, or more specifically deep learning, has been adapted to classify diffraction patterns (Souza *et al.*, 2019 ▸; Ke *et al.*, 2018 ▸; Becker & Streit, 2014 ▸). Intuitively, deep neural models can encode features of diffraction patterns, including background and Bragg peaks, to classify them into hit or miss categories in order to reduce the data.

In this work, our goal is to build a data reduction method for serial crystallography that is computationally cheaper and less reliant on parameters. Thus, we propose a novel mechanism to detect and identify key points representing ‘some’ Bragg peaks in diffraction patterns. Specifically, we use the oriented FAST and rotated BRIEF (ORB) handcrafted method to extract key points representing Bragg peaks, inheriting good performance numbers and low computational cost (Rublee *et al.*, 2011 ▸). Afterwards, we train a multilayer perceptron (MLP) on these extracted features to distinguish between hits and misses in diffraction patterns, resulting in data reduction. Our results on synthetic and experimental data indicate that the extracted key points encode Bragg peak information and thus provide suitable input for the classification task. Our main technical contributions are as follows:

(i) A handcrafted method based on ORB features to extract key points that contain Bragg peak information and then train an MLP to classify diffraction patterns for data reduction.

(ii) A novel study to compare various handcrafted feature extractors along with machine learning classifiers on synthetic and real experimental data. Our study reveals that ORB features effectively encode Bragg peaks and are robust against anomaly scenarios compared with other state-of-the-art feature extractors.

(iii) An automated pipeline based on ORB features to label diffraction patterns for supervised machine learning methods.

## 2. Related work

There are two broad ways of reducing serial crystallography data: compressing images and rejecting bad images. (Naturally, both of these methods can be applied to a data set.) In turn, rejecting bad images can be done in two ways: using peak finding methods based on statistical frameworks, or machine learning. Typically, peak finding methods accept or reject a diffraction pattern by finding Bragg peaks and counting the total number of peaks. Machine learning techniques extract features and classify images by learning from training data, without necessarily finding peak locations.

Data compression is a procedure in which the size of the data set is reduced by re-encoding the data to use fewer bits of storage than the original data. Compression algorithms can be lossless (information is fully preserved and compression is achieved purely by removing statistical redundancy) or lossy (some information is lost, reducing the resolution of the data).

In experiments at synchrotrons, photon-counting detectors are typically used, giving images where each pixel records the integer number of photons that hit it during the exposure. Under these conditions, good compression ratios can be achieved using lossless compression methods. However, in experiments at FELs all the photons arrive in a pixel simultaneously, which means that integrating detectors are required, where each pixel measures the total energy deposited with a certain amount of electronic noise. Under these conditions, lossy compression is typically required for reasonable compression ratios.

Leonarski *et al.* (2020 ▸) investigated the efficacy of the SZ compressor technique (Di & Cappello, 2016 ▸) on the data generated by a JUNGFRAU detector. They demonstrated that, while vast compression ratios (up to 168×) are possible with SZ 2.1.7 (compression ratios relative to float32), this can result in poor quality, particularly in regions with small local intensity changes. Therefore, their method can only utilize relatively small error bounds to guarantee the reconstructed data quality, which results in moderate compression ratios (up to 10×).

A novel data compression method developed for use in upcoming high-data-rate facilities such as LCLS-II-HE has been presented by Underwood *et al.* (2022 ▸). This method is region of interest binning with SZ lossy compression (ROIBIN-SZ), a novel parallel and accelerated compression technique that splits the dynamically selected protection of key regions with lossy compression of background information.

Techniques for detecting Bragg peaks in diffraction patterns are a crucial building block in both rejecting bad images and analysing data. Peak finding methods employ carefully crafted threshold mechanisms based on statistical frameworks to separate Bragg peaks from the background signal (Parkhurst *et al.*, 2016 ▸; Hadian-Jazi *et al.*, 2017 ▸, 2021 ▸). For example, Li & Zatsepin (2018 ▸) used a simple global threshold mechanism to separate the background signal from the Bragg peaks. Although the mechanism is straightforward, its effectiveness is often highly dependent on the correct tuning of many input parameters.

In turn, various tools have been built that make use of these peak finding methods. For example, the *Cheetah* software suite (Barty *et al.*, 2014 ▸) has been developed to filter diffraction patterns to give a significant reduction in the data. It employs key data quality metrics such as the number of Bragg peaks to retain samples with a high probability of being usable for structure determination. The principal parts of *Cheetah* perform detector corrections, detect Bragg peaks from the diffraction pattern, sort crystal diffraction patterns, and convert them into a facility-independent format for further analysis such as indexing and determining the orientation of crystal planes. Detector corrections include specifying and flagging bad and dark pixels of each module from the detector and applying individual gain corrections for each pixel. When the corrected image is obtained, *Cheetah* explores potential Bragg peaks in the diffraction image using a threshold mechanism called the Peakfinder8 algorithm. This algorithm finds all Bragg peaks with a size of more than *n*_min_ but fewer than *n*_max_ connected pixels with intensities above a radially dependent threshold, which is computed from the averaged background intensity. If the number of found peaks with an adequately high signal-to-noise ratio surpasses a certain minimum number *n*_peaks_, the image is considered as a hit image. Finally, reduced data are output in a facility-independent HDF5 format, enabling the use of downstream analysis software suites such as *CrystFEL* (White *et al.*, 2012 ▸) which are typically employed to view, index, integrate, merge and evaluate the quality of the diffraction data. More recently, the *OnDA* (Mariani *et al.*, 2016 ▸) software suite provides real-time monitoring of X-ray diffraction data along with experimental conditions. While peak finding methods and tools can successfully handle, process and analyse diffraction data, they require carefully curated input parameters from experts.

At XFELS in particular, where experimental teams can involve many researchers, scientists and users, experimental results need to be communicated effectively to all groups, including sample preparation units, beamline scientists, sample injection groups and data analyst engineers. All the data processing must also be automated to let scientists, researchers and users spend time investigating the experimental data specifically, instead of being distracted by the techniques and mechanics of submitting many jobs and monitoring their state. The *cctbx.xfel* GUI (Brewster *et al.*, 2019 ▸), which is a graphical user interface application, open-source software, and part of the *cctbx* and *DIALS* software packages (Grosse-Kunstleve *et al.*, 2002 ▸; Winter *et al.*, 2018 ▸), allows crystallography scientists and researchers to move rapidly through all steps of data reduction, especially for serial crystallography.

As mentioned, rejecting bad images can also be done using machine learning. The past decade has seen unprecedented breakthroughs for a wide variety of computer vision tasks based on the subset of machine learning methods referred to as deep learning (LeCun *et al.*, 2015 ▸). Other fields such as text (Mikolov *et al.*, 2013 ▸), audio (Nagrani *et al.*, 2017 ▸; Saeed *et al.*, 2021 ▸) and more recently serial crystallography have successfully used deep learning (Becker & Streit, 2014 ▸). Specifically, Ke *et al.* (2018 ▸) adapted a deep neural model with a structure similar to that of AlexNet (Krizhevsky *et al.*, 2012 ▸) to encode Bragg peaks to classify crystallographic diffraction patterns. Similarly, Souza *et al.* (2019 ▸) used a residual neural network with 50 layers (ResNet-50) dubbed DeepFreak to classify patterns from both synthetic and real diffraction data. In addition, they presented a comparative study of deep neural models and a few computer vision feature extractors, with the former outperforming the latter. It is well understood in the computer vision community that deep neural models often outperform traditional computer vision feature extractors on image classification tasks (Sharif Razavian *et al.*, 2014 ▸). However, deep neural models are computationally expensive.

In contrast to existing methods, our work differs in following respects: (i) the proposed pipeline does not require carefully selected parameters to detect Bragg peaks to accept or reject diffraction patterns in order to reduce serial crystallography data, and (ii) we have made a novel comprehensive study to compare various handcrafted feature extractors along with machine learning classifiers on both synthetic and real experimental data. We observe that the existing literature lacks a comprehensive comparative study using serial crystallography data.

## 3. Proposed pipeline

**Figure 2:**
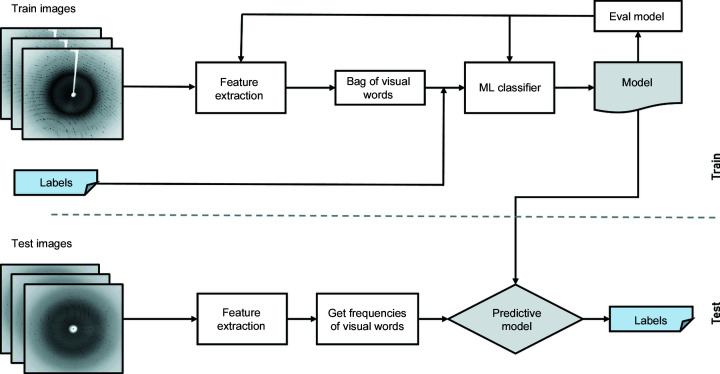
The proposed computation pipeline that extracts key points to train and test a machine learning classifier. It extracts key points from diffraction patterns representing Bragg peaks with a feature extractor. Afterwards, similar features are grouped together with the ‘bag of visual words’ method. Finally, a machine learning classifier is trained with the features.

Our goal is to classify X-ray crystallography diffraction data into hit (the X-ray beam hits a crystal) and miss (the X-ray beam does not hit a crystal) categories to reduce the data, using machine learning. Thus, we propose a pipeline with four main components consisting of feature extraction, representing extracted features using the bag of visual words (BoVWs) method, diffraction pattern labelling and classification with machine learning (Fig. 2 ▸). The pipeline contains a feature extractor to encode key points representing Bragg peaks (Section 3.1). We compared several well known feature extraction methods from computer vision in order to find the best candidate. BoVWs were then created using *K*-mean clustering to generate the codebooks and treating features as words (Section 3.2). In addition, the pipeline includes an automated labelling mechanism using ORB key points and descriptors (Rublee *et al.*, 2011 ▸) along with a threshold value to label diffraction patterns (Section 3.3). Finally, we use a machine learning classifier to categorize the diffraction patterns, comparing the performance of four supervised machine learning models: MLP, support vector machine, random forest and naïve Bayes (Section 3.4).

### 3.1. Handcrafted feature extractors

Handcrafted feature extractors encode the content of an image using a number of points or neighbourhoods. Typically, the encoding can vary from raw values to histograms of gradients and point-wise comparisons. In ground-breaking work, Schmid & Mohr (1997 ▸) presented local features which are computed at automatically detected interest points for an image retrieval task. Since then, handcrafted features have made remarkable progress, handling various computer vision tasks (Dalal & Triggs, 2005 ▸; Lowe, 2004 ▸; Perronnin *et al.*, 2010 ▸). In this work, we studied several well known feature extractors from the computer vision community (ORB, SIFT, BRIEF, Haris, SURF, FAST, Shi-Tomasi, HOG) to extract features from diffraction patterns. Our experimental results show that the SIFT, SURF and Shi-Tomasi algorithms extract feature vectors even from the background of the image and not only from the Bragg peaks. The BRISK, BRIEF and Haris algorithms extract features from Bragg peaks but the number of detected key points is lower than with ORB. We also implemented the histogram of oriented gradients (HOG) feature descriptor. HOG counts events of gradient orientation in a specific piece of an image or region of interest. The HOG feature extractor focuses on the structure of the object and extracts many features from the background of the image.

As shown in Section 4.3.1, the ORB feature extractor produces the best results in both the number of detected key points and the matching of key points to Bragg peaks; therefore, we will describe this particular method in more detail.

#### 3.1.1. Oriented FAST and rotated BRIEF

**Figure 3:**
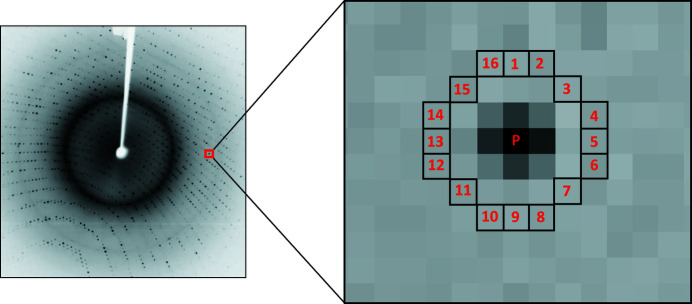
The FAST key point detection mechanism. A pixel *p* is compared with 16 neighbouring pixels in a circle formulation. If more than eight pixels are darker or brighter than *p* then it is selected as a key point.

ORB is a well known robust local feature detector and is extensively applied in computer vision tasks (Rublee *et al.*, 2011 ▸). It is based on features from the accelerated and segments test (FAST) key point detection approach and a modified version of the binary robust independent elementary features (BRIEF) key point descriptor algorithm, inheriting reliable performance and low cost (Rublee *et al.*, 2011 ▸). For each pixel *p* in an image, the FAST algorithm compares the brightness of *p* with the neighbouring 16 pixels that are in a circle around that pixel. Pixels within the circle are then ordered into three categories (lighter than *p*, darker than *p* or similar to *p*; Fig. 3 ▸). If more than eight pixels are brighter or darker than *p* then it is picked as one key point. Thus key points detected by the FAST approach provide us with information on the position to determine the edges in an image.

**Figure 4:**
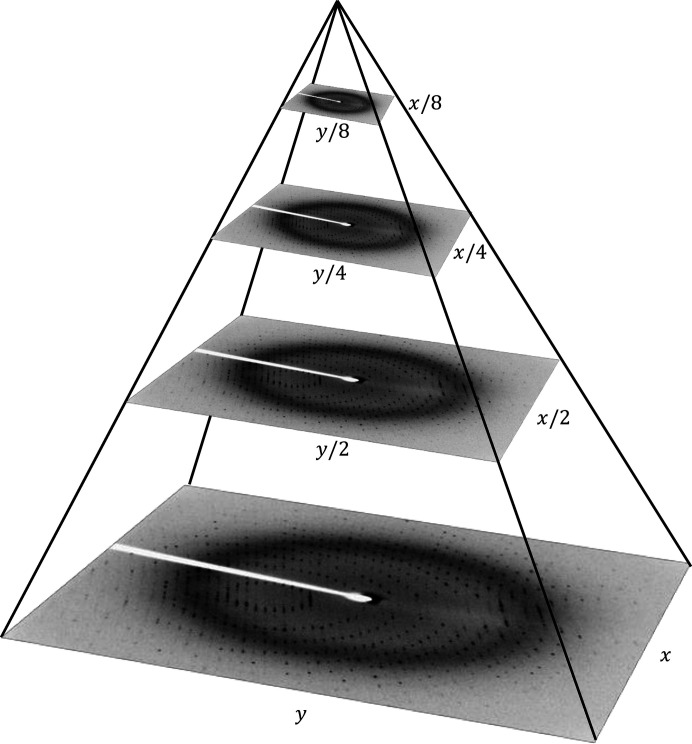
A diffraction pattern multiscale image pyramid with width *x* and height *y*, including various versions of downsampled input diffraction patterns from which key points can be extracted.

Nevertheless, FAST features are without orientation and multiscale components. Therefore, the ORB feature extraction method applies a multi-scale pyramid. A pyramid is a multiscale illustration of a particular input sample with several series of images at varying resolutions of the original image. In this case, each level in the pyramid is downsampled from the previous level. Once ORB has built a pyramid, it employs the FAST approach to identify key points in the image. By detecting all potential key points at each level of the pyramid, ORB efficiently discovers key points on a varying scale, making it scale invariant. Fig. 4 ▸ shows an example of a multi-scale diffraction pyramid.

**Figure 5:**
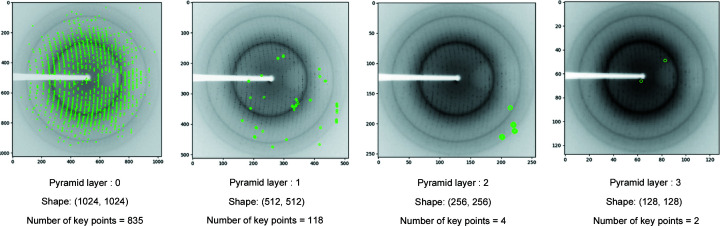
Detected key points at different pyramid levels of the ORB feature detection (green circles show detected key points).

Experimental results show that most of the key points are detected on an earlier level of the pyramid (Fig. 5 ▸). Therefore, in order to have a faster computation time, we have extracted key points in just the first layer of the pyramid and ignored the key point detection in the other layers.

In the next step, ORB allocates an orientation for each detected key point depending on how the levels of intensity shift around that key point. It employs intensity centroiding (Rosin, 1999 ▸) in order to detect intensity variations. The intensity centroid estimates that a key point’s intensity is offset from its centre and this vector may be used to assign an orientation. First, the moments of a patch are defined as

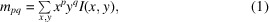

where *x* and *y* are pixel coordinates and *I*(*x*, *y*) is the grey value of the corresponding pixel. With these moments, ORB finds the centroid, the ‘centre of mass’, of the patch as

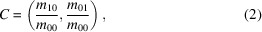

where the zeroth moment (*m*_00_) is the mass of the image block and the first moment (*m*_10_, *m*_01_) is the centroid of the image block.

We can create a vector from the corner’s centre *O* to the centroid, 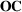 . The orientation of the patch is then given by

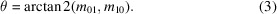

Once the orientation of the patch has been computed, it is possible to rotate it to a canonical rotation and then compute the descriptor, resulting in rotation invariance. The key points detected with the FAST method are given to BRIEF, converting them into a binary feature vector (also known as binary feature descriptor).

The BRIEF feature describes a diffraction pattern by a set of key points, with each one being described by a feature vector consisting of 128–512 bits. BRIEF uses a Gaussian kernel for smoothing the diffraction pattern to avoid the descriptor being sensitive to high-frequency noise. Then a random pair of pixels are picked from a pre-specified neighbourhood, which is a square of some pixel width and height, known as a patch, around each computed key point. The first pixel in the randomly picked pair is drawn from a Gaussian distribution by a standard deviation or spread of sigma centred around the key point, and the second pixel is drawn from a Gaussian distribution centred around the first pixel by a standard deviation or spread of two sigma (Rublee *et al.*, 2011 ▸). If the first pixel is brighter than the second one, the bit is set to 1; otherwise it is set to 0. Again BRIEF chooses a random pair of pixels and indicates the value to them. For a 128 bit vector, it repeats 128 times and generates one vector for each detected key point from the input. The same random pairs are used for each key point, so that identical features will produce identical feature vectors.

Mathematically, the method can be described as follows. Consider a smoothed image patch *p*. A binary test τ is defined by

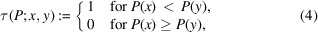

where *P*(*x*) is the intensity of *P* at point *x*. A vector of *n* binary tests is defined by a feature as

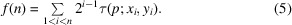




Nevertheless, the basic version of BRIEF described thus far is not robust against rotation variation; if two otherwise identical features are rotated with respect to each other by more than a few degrees, they will produce different feature vectors. ORB uses rotation-aware BRIEF (rBRIEF) in order to make it robust against rotation variation. In this method, for each given feature the set *S* of *n* binary tests at position (*x*_*i*_, *y*_*i*_) is defined as a 2 × *n* matrix,

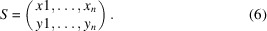

It uses the patch orientation θ and the corresponding rotation matrix *R*_θ_ to construct a steered version *S*_θ_ of *S*,

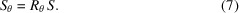

The rBRIEF operator is now

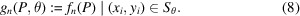

Finally, the discretization of the angle is formed at increments of 2π/30 (12°) and a lookup table of precalculated BRIEF patterns is generated. While the orientation of each key point θ is consistent across views, the correct set of points *S*_θ_ will be employed to compute its descriptor.

### 3.2. Bag of visual words

In natural language processing, the bag of words method represents text information with a simple histogram of word frequencies. Similarly, BoVWs takes features extracted from an image and creates a histogram of different features (Sivic & Zisserman, 2003 ▸; Csurka *et al.*, 2004 ▸). To do this, however, a codebook that groups features into different categories must be created. A codebook is recognized as a representation of various similar patches. In our pipeline, we used ORB features to generate the codebook for the BoVW model. In order to generate the codebook, we applied the mini batch *K*-means clustering approach (Sculley, 2010 ▸) over features (ORB) and the visual words in the codebook are defined by the centres of the learned clusters. Therefore, each patch in the image is mapped to a specific codebook within the clustering process and, finally, the image is represented by a histogram of visual words. The generated histograms are then used to train a machine learning classifier.

**Figure 6:**
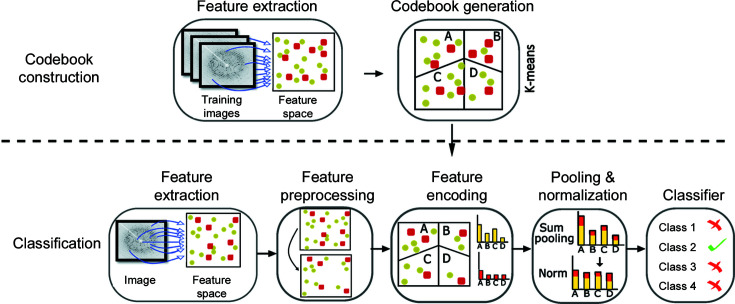
An illustration of the bag of visual words approach. The first row shows the process of learning a vocabulary of visual words by computing the ORB descriptor and clustering the collection of descriptors into groups whose centres will define the visual words by *K*-means clustering. The second row shows how we use the visual word tree. Given a diffraction pattern, we compute the ORB descriptors. For each descriptor we then find the closest cluster centre and increment the frequency count for that visual word. The result is a histogram of visual word counts.

Mini batch *K*-means clustering is a faster version of the *K*-means algorithm which can be used instead of the latter when clustering on huge data sets. It makes random batches of data that are stored in memory, and then a random batch of data is collected on each iteration to update the clusters. We consider *n*_clusters_ = 5*n*_classes_, where *n*_clusters_ is the number of clusters, known as the size of the codebook, and *n*_classes_ is the number of classes in the data set. We also consider *b* = *S*/3 as the batch size, where *S* is the length of all feature vectors for all classes, and int_size_ = 3*b* as the initial size of clustering, and these are inputs for the mini batch *K*-means clustering. Fig. 6 ▸ shows a block diagram for the generation of BoVWs.

### 3.3. Diffraction pattern labelling

**Figure 7:**
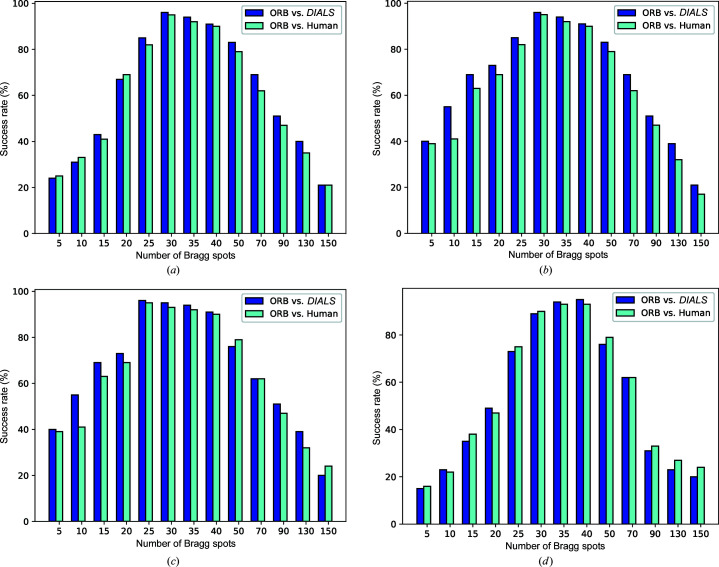
The success rates of the ORB key points compared with a human annotator and the *DIALS* automatic spot finder for each of the four data sets studied here. (*a*) L498, (*b*) LN84, (*c*) LN83 and (*d*) LO19.

It is vital to build a high-quality labelled data set for supervised machine learning (Russell & Norvig, 2002 ▸). Typically, supervised machine learning methods infer a function from labelled training data consisting of a set of training samples. Due to the nature of SFX experiments, it is a challenging task to label large-scale unlabelled diffraction patterns. Typically, diffraction patterns are labelled with the help of human annotators (Ke *et al.*, 2018 ▸) or an automated mechanism such as the diffraction integration for advanced light sources (*DIALS*) spot finder (Parkhurst *et al.*, 2016 ▸). Our proposed labelling method extracts ORB key points from a diffraction pattern, capturing some of the Bragg peaks. Specifically, the method extracts key points by considering the pixel brightness around a given area. Afterwards, the detected key point is converted into a vector typically known as a descriptor. Intuitively, the ORB key point detector works like a peak finder, so we use the detector to count the number of Bragg peaks and employ a threshold value τ to label a diffraction pattern as a hit or miss. Given the nature of SFX experiments, the number of Bragg peaks can vary between diffraction experiments, so the threshold value τ is data set specific. To find the optimal value for each data set, we varied the threshold value (τ) and compared the results with those from human annotation and the *DIALS* spotfinder. For experimental data, we selected four diverse XFEL experimental data sets (denoted L498, LN84, LN83 and LO19), containing diffraction patterns from different detectors, samples and delivery methods (see Section 4.1 and Table 1). We found τ = 30, 30, 25 and 40 for L498, LN84, LN83 and LO19, respectively (Fig. 7 ▸).

### 3.4. Classification

X-ray diffraction pattern classification is a two-step process. In the first step, the ORB feature extraction technique is applied to obtain key points, feature vectors and descriptors from labelled patterns. In the second step, a machine learning algorithm is trained to classify images into data categories. We compared four supervised classifiers, namely MLP, support vector machine (SVM), random forest (RF) and naïve Bayes (NB).

#### 3.4.1. Multilayer perceptron

This is a feed-forward neural network that consists of a unidirectional network distributed in a set of input and output layers (Ramchoun *et al.*, 2016 ▸). It consists of at least three layers: an input layer to receive the input feature vectors, one or more hidden layers allowing the capability to learn nonlinear models (Lippmann, 1987 ▸; Duda *et al.*, 2000 ▸), and an output layer to make a prediction (Lu & Weng, 2007 ▸).

For hyperparameter tuning, we used the *GridSearchCV* tool from the *Sklearn* library (Pedregosa *et al.*, 2011 ▸). We experimented with different sizes of hidden layers, activations (tanh, ReLU), solvers (SGD, adam), alphas (0.0001, 0.05) and learning rates (constant, adaptive). After hyperparameter tuning, the parameters were as follows: stochastic gradient descent (SGD) was used for weight optimization; seven hidden layers were used, with *L* neurons in each layer where *L* = {50, 30, 20, 20, 20, 30, 50}; the activation function was the rectified linear unit (ReLU) which returns 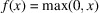 ; and the regularization term was 1 × 10^−5^. The number of input neurons is different for each data set and it is equal to the number of clusters in the mini batch *K*-means clustering algorithm used in performing the BoVWs step.

#### 3.4.2. Support vector machine

This is a supervised machine learning technique that can be used for either image classification or regression problems. The SVM classifier applies a method called the kernel trick, which separates data points using a hyperplane with the largest amount of margin. In other words, the kernel takes low-dimensional input data and transforms it into a higher-dimensional space in order to convert nonseparable problems to separable problems by adding more dimensions. Additionally, the SVM performs good generalization, which avoids overfitting (Duda *et al.*, 2000 ▸). In our work, we used the radial basis function (RBF) kernel with a γ parameter of γ = 0.001. The RBF kernel is one of the most common kernel functions used in SVM classification. It has the form

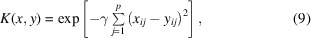

where γ is a tuning parameter which accounts for the smoothness of the decision boundary and controls the variance of the model. Intuitively, the value of γ determines how far the influence of a particular training sample reaches, with low values indicating ‘far’ and high values indicating ‘close’.

#### 3.4.3. Random forest classifier

RF is one of the most common supervised machine learning techniques and is based on decision-tree algorithms. Like SVM, RF is also used to solve regression and image classification problems. It uses ensemble learning, which is a method that mixes many classifiers to solve complex problems. The RF technique formulates the result on the basis of the predictions of the decision trees. It is a meta estimator that implements a number of decision-tree classifiers on different sub-samples of the input data, and employs averaging to increase the predictive accuracy and control overfitting. Increasing the number of trees also increases the precision of the outcome. Generally, for a classification problem, the output of the RF is the class selected by most trees. We have used the RF classifier because it is more accurate than the decision-tree approach and it can provide a reasonable prediction without any need for hyper-parameter tuning (Breiman, 2001 ▸). In this paper, we considered *n*_estimators_ = 100 as the number of trees in the forest and the ‘Gini impurity’ as a function to measure the quality of a split as follows: 

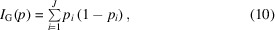

where *p_i_* is the probability of a certain classification *i*, as per the training data set.

#### 3.4.4. Naïve Bayes classifier

This is a straightforward and powerful supervised learning technique for classification tasks. NB classifiers are probabilistic classifiers based on Bayes’ theorem which assume statistical independence between the features of the data set. The primary idea of the NB model is that each class has its own distribution over the codebooks where the distributions of each class are noticeably different. Classification is done by finding the class with the highest posterior probability (Lu & Weng, 2007 ▸). This approach is known as maximum *a posteriori* (MAP) and is defined as

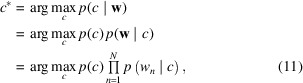

where *c* refers to classes, *w* refers to features and the algorithm will pick the class *c* with the highest probability.

In this paper, we have used the Gaussian NB classifier, where the assumption is that data from each class are drawn from a simple Gaussian distribution. As we demonstrated in Section 3.2, it is possible to generate a simple model by assuming that the ORB extracted features for each class are described by a Gaussian distribution with no covariance between feature spaces. This model can be fitted simply by using the mean and standard deviation of the features inside each class, which are all the parameters needed to determine such a distribution. In this case the formula for the conditional probability changes as follows:

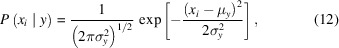

where σ is the standard deviation and μ is the mean for *x*_*i*_.

## 4. Experimental details

In this section, we discuss the experimental data along with the implementation details of our pipeline. In addition, we provide experimental results and discussions of various feature extractors used on the classification task.

### 4.1. Data sets

In our experiments, we used both synthetic and real experimental serial crystallography data to evaluate the pipeline. The synthetic data set named DiffraNet is generated with the *nanoBragg* simulator using a single-crystal structure (Souza *et al.*, 2019 ▸). Variations in image quality are achieved by varying the X-ray beam intensity or by modelling imperfections in the crystal by breaking it up into smaller crystals. Other parameters include sources of background noise and the crystal orientation. It consists of 25 000 diffraction patterns with an image size of 512 × 512, divided into five classes (Blank, No crystal, Weak, Good, Strong) to perform the classification task. In addition, we created another set named DiffraNetHM where the No crystal and Weak classes are merged into misses while the other classes are grouped into hits, to make it consistent with real experimental data sets.

For experimental data, we selected four diverse experimental data sets to reflect different imaging detectors, beam energies and sample delivery methods and to include crystals with different space groups and unit-cell parameters. Table 1 ▸ lists these protein serial crystallography diffraction data sets, which were collected on the Coherent X-ray Imaging (Boutet *et al.*, 2016 ▸) and Macromolecular Femtosecond Crystallography (Bostedt *et al.*, 2016 ▸) instruments at the Linac Coherent Light Source (Bostedt *et al.*, 2016 ▸) (LCLS, Menlo Park, California, USA). Recently, Ke *et al.* (2018 ▸) unpacked the first 2000 images from the native LCLS data format and converted them to a four-byte integer HDF5 format for further study. We used the same diffraction patterns and evaluation protocols in our experiments.

### 4.2. Implementation details

Our pipeline consists of feature extractors and machine learning components. We evaluated the performance of the pipeline with standard classification evaluation metrics, *i.e.* accuracy, precision, recall and *F*1 scores. Experiments were performed with the Python 3.6, *OpenCV* (https://opencv.org/) and *scikit-image* (https://scikit-image.org/) libraries, on an Intel Core i5 CPU operating at 2.21 GHz, with 1 MB L2 cache, 8 MB L3 cache and 16 GB RAM.

### 4.3. Experimental results

#### 4.3.1. Feature extractors – qualitative and quantitative results

**Figure 8:**
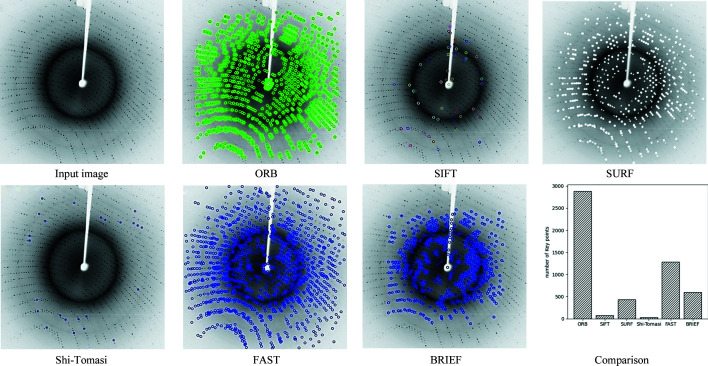
Key point detection of various feature extractors. Key points indicate that the ORB extractor extracts regions roughly equal to the number of Bragg peaks in the diffraction pattern.

Generally, features obtained from an extractor are used to train the machine learning model, so it is essential to extract key regions from a diffraction pattern representing Bragg peaks. In other words, features with Bragg peaks will be essential to train a robust machine learning classifier. In this section, we visualize key regions of a diffraction pattern representing Bragg peaks using various feature extractors (Fig. 8 ▸). Our qualitative results indicate that the ORB feature extractor extracts key points corresponding to the Bragg peaks in the diffraction pattern. Other feature extractors extract either too few or too many key points from the background, which is not suitable for training a machine learning model. Quantitatively, the results show that the ORB extractor is superior to the others. Table 2 ▸ shows a comparison of the feature extractors used here in terms of memory, execution time and classification performance.

**Figure 9:**
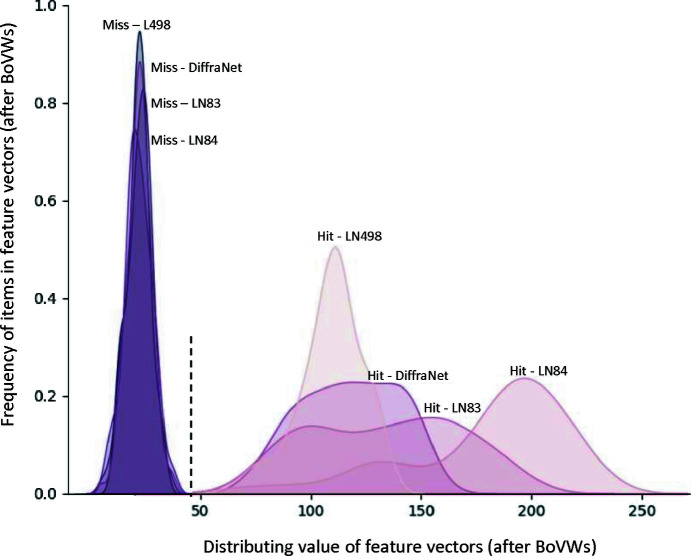
Feature distributions of various serial crystallography data sets.

In addition, we visualized the ORB features extracted from the diffraction patterns, with each pattern describing a point in the feature or embedding space (Fig. 9 ▸). It is evident that hit and miss classes are well separated by their ORB features, indicating the discriminative nature of the method.

#### 4.3.2. Robustness against anomalies

**Figure 10:**
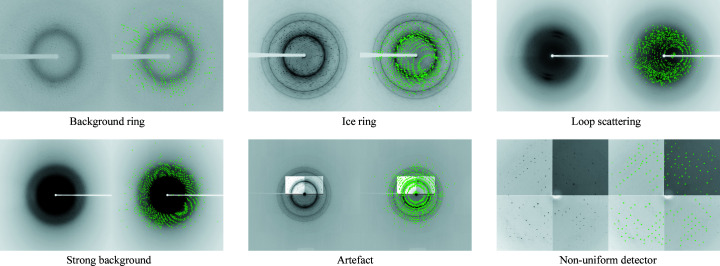
ORB feature extraction from Bragg peaks with various anomalies.

X-ray images can contain other features aside from the crystal X-ray diffraction we want to measure. Some of these features are digital artefacts produced by the detector itself, whereas others are produced by X-rays scattering from other material in the experiment. Some kinds of X-ray diffraction pattern anomalies are water scattering (background ring), loop scattering, non-uniform detector responses, ice rings, low crystal signal-to-noise ratio (strong background) and digital artefacts (Czyzewski *et al.*, 2021 ▸). These anomalies can change the regular shape of a diffraction pattern. Our qualitative results show that the ORB feature extractor is robust against anomalies. Fig. 10 ▸ shows that ORB extracts features from Bragg peaks with diffraction patterns containing different anomalies.

#### 4.3.3. Diffraction pattern labelling method

Our labelling method automatically labels diffraction patterns into hit or miss classes to train supervised machine learning models. The labelling method extracts key points from a diffraction pattern with the ORB detector. Afterwards, a threshold value is selected to label a pattern into one of the classes (see Fig. 7 ▸). We evaluated our method with two ground-truth labels collected from a human expert and the automated *DIALS* spot finder (Ke *et al.*, 2018 ▸). Table 3 ▸ shows the performance of our labelling method along with that of the human expert and *DIALS* spot finding ground-truth label for four data sets. Although our labelling method requires a manual selected threshold value, the results indicate that it can be reliably used to label offline diffraction patterns for supervised machine learning models.

#### 4.3.4. Classification results

In this section, we provide details of the classification performance using the MLP, SVM, NB and RF classifiers trained on ORB features.

The entire feature extraction and classification pipeline was performed on an Intel Core i5-10310U CPU (consisting of four-core chips, 1.7 GHz) with 6 MB cache and 16 GB RAM, running Microsoft Windows 10, with code programmed in Python. In all our classification experiments, we used different images for training and testing to avoid overfitting. In our first set of experiments, we looked at each data set independently. We trained using 80% of the images from the data set and then tested using the remaining 20%, employing the fivefold cross-validation method to improve statistics. Table 4 ▸ shows the classification results for *F*1 score, Precision, Recall and Accuracy with the same training and testing strategy on both synthetic and real experimental data, where

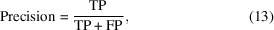




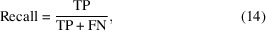




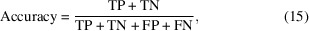




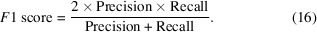

TP is defined as true positive, TN as true negative, FP as false positive and FN as false negative.

These results clearly demonstrate that the ORB+MLP method produces superior performance across all data sets. In addition, ORB+MLP produces performance above 90%, with the exception of data set L498. The L498 data set contains a relatively small number of Bragg peaks compared with the other data sets, which makes the classification task more challenging. A similar conclusion was drawn by Ke *et al.* (2018 ▸).

Table 5 ▸ shows a comparison of processing times for the various components of our proposed method and the CNN base image classification system (Ke *et al.*, 2018 ▸).

In the second type of classification experiments, we performed cross-data training and testing, where for each pair of data sets we first trained on one data set and then tested on the other. This approach tests the possibility of deploying our pipeline for real-time classification of new experimental data that presumably make use of similar sample delivery systems and detectors to those used for the training data. Table 6 ▸ shows the results for training and testing cross data sets, resulting in a drop in performance due to the domain gap. In addition, Fig. 9 ▸ shows the variation in embedding space for the hit classes from four different data sets (LN84, LN83, L498 and DiffraNet). In other words, differences in experimental features such as the sample delivery method and detector may affect the performance of our proposed pipeline.

**Figure 11:**
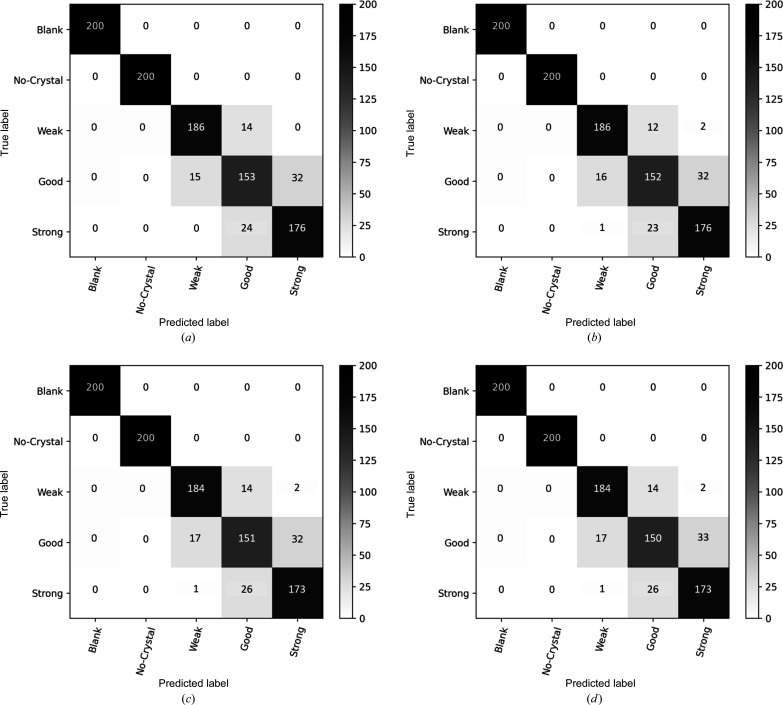
Confusion matrices for the classification DiffraNet data set with five classes. (*a*) ORB+MLP, (*b*) ORB+SVM, (*c*) ORB+RF and (*d*) ORB+NB.

Finally, we compared the ORB features with various machine learning classifiers on the DiffraNet data set. We tested the machine models to classify the diffraction patterns into five classes, namely Blank, No crystal, Weak, Good and Strong. Fig. 11 ▸ shows the confusion matrices for various machine learning models on DiffraNet. Most of the mis­classification cases happen between neighbouring classes such as Weak, Good and Strong.

## 5. Conclusions and future work

In this work, we have investigated the practicability of using handcrafted feature extraction methods with a novel pipeline for diffraction patterns obtained in serial crystallography experiments. Our experimental results show that ORB feature extraction is the best candidate. The major benefit of the ORB feature extractor is that it does not require a precise characterization of the Bragg peaks or of the many undesirable artefacts present in the diffraction patterns.

We observed that a classifier trained on one data set cannot necessarily be applied to data collected with different experimental settings. Although it is desirable to train a classifier that can be used on all experiments, it is challenging to collect a large-scale data set representing various experimental settings. Therefore, we want to take advantage of domain adaptation methods to bridge the performance drop due to changes in the experimental setting (Morerio *et al.*, 2017 ▸).

## Figures and Tables

**Table 1:** Experimental data

LCLS data set (proposal, run)	Incident energy (eV)	Protein	Space group, unit cell (Å)	Instrument	Sample delivery	Detector
L498, 27	9773	Thermolysin	*P*6_1_22, *a* = 93, *c* = 130	CXI	MESH	CSPAD
LN84, 95	9516	Photosystem II	*P*2_1_2_1_2_1_, *a* = 118, *b* = 223, *c* = 311	MFX	Conveyor belt	Rayonix
LN83, 18	9498	Hydrogenase	*P*2_1_2_1_2_1_, *a* = 73, *b* = 96, *c* = 119	MFX	Conveyor belt	Rayonix
LO19, 20	9442	Cyclophilin A	*P*2_1_2_1_2_1_, *a* = 42, *b* = 52, *c* = 88	MFX	Liquid jet	Rayonix

**Table 2:** Memory and time for each extractor representing a single feature Classification performance is included with MLP on the LN84 data set.

Descriptor	SIFT-128	SURF-64	Shi-Tomasi	BRIEF	ORB	FAST
Memory (bytes)	128	64	64	32	32	16
Time (ms)	2.776	1.024	0.088	0.224	0.179	0.092
Accuracy (%)	51.66	68.49	12.78	59.18	96.5	41.38

**Table 3:** Success rate (%) of ORB labelling compared with the *DIALS*- and human-predicted labels

		L498 (τ = 30)		LO19 (τ = 40)		LN84 (τ = 30)		LN83 (τ = 25)	
	ORB label	Hit	Miss	Hit	Miss	Hit	Miss	Hit	Miss
*DIALS* label	Hit	95.19	4.81	96.12	3.88	96.94	3.06	96.63	3.37
	Miss	7.28	92.72	3.59	96.41	6.83	93.17	5.36	94.64
Human label	Hit	94.21	5.79	95.49	4.51	95.07	4.93	95.18	4.82
	Miss	4.53	95.47	6.29	93.71	8.42	91.58	9.81	90.19

**Table:** 

ORB+MLP							ORB+SVM					
Data set	Labels	*F*1 (%)	Precision (%)	Recall (%)	Accuracy (%)		Data set	Labels	*F*1 (%)	Precision (%)	Recall (%)	Accuracy (%)
L498	Human	90.26	89.16	88.27	90.12		L498	Human	90.08	90.40	87.18	88.72
	*DIALS*	90.43	89.59	89.69	90.83			*DIALS*	90.32	91.41	89.00	89.17
	ORB	89.42	88.54	87.52	89.71			ORB	89.12	90.36	86.00	88.50
LO19	Human	92.23	93.45	91.72	92.59		LO19	Human	85.61	87.66	83.49	85.71
	*DIALS*	92.65	93.97	91.88	92.82			*DIALS*	85.98	87.71	83.92	85.88
	ORB	91.91	92.85	91.00	92.00			ORB	85.12	87.36	83.00	85.50
LN84	Human	96.71	97.46	96.73	96.22		LN84	Human	94.31	94.44	94.39	94.14
	*DIALS*	96.78	97.36	96.49	96.97			*DIALS*	94.25	94.49	94.77	94.18
	ORB	96.48	96.96	96.0	96.5			ORB	93.53	93.06	94.0	93.5
LN83	Human	93.73	94.97	93.38	93.79		LN83	Human	92.14	93.39	91.54	92.50
	*DIALS*	95.11	94.96	92.77	94.38			*DIALS*	91.40	94.84	92.00	93.50
	ORB	93.40	94.84	92.00	93.50			ORB	91.83	93.75	90.00	92.00
DiffraNet	Simulated	97.51	97.02	98.00	97.50		DiffraNet	Simulated	95.52	95.04	96.00	95.50

**Table:** 

ORB+RF							ORB+NB					
Data set	Labels	*F*1 (%)	Precision (%)	Recall (%)	Accuracy (%)		Data set	Labels	*F*1 (%)	Precision (%)	Recall (%)	Accuracy (%)
L498	Human	80.66	80.50	81.54	80.72		L498	Human	82.09	82.71	81.39	82.18
	*DIALS*	80.99	80.79	81.73	80.81			*DIALS*	82.45	82.91	81.68	82.33
	ORB	80.59	80.19	81.0	80.5			ORB	81.81	82.65	81.0	82.0
LO19	Human	82.94	84.39	80.12	82.73		LO19	Human	81.36	82.65	80.17	81.83
	*DIALS*	83.12	84.78	80.42	82.84			*DIALS*	81.88	82.92	80.18	81.91
	ORB	82.05	84.21	80.0	82.5			ORB	81.21	82.47	80.0	81.5
LN84	Human	88.93	90.55	87.52	89.51		LN84	Human	89.77	89.71	90.00	90.04
	*DIALS*	89.38	90.87	87.47	89.57			*DIALS*	89.91	89.72	90.00	90.11
	ORB	88.77	90.62	87.0	89.0			ORB	89.55	89.10	90.0	89.5
LN83	Human	92.18	92.53	92.42	92.12		LN83	Human	83.18	88.72	77.71	82.97
	*DIALS*	92.40	92.84	92.73	92.50			*DIALS*	83.41	88.97	77.92	83.06
	ORB	92.0	92.0	92.0	92.0			ORB	82.52	88.54	77.27	82.85
DiffraNet	Simulated	94.58	93.20	96.00	94.50		DiffraNet	Simulated	92.07	91.17	93.00	92.00

**Table 5:** Processing time (ms) for various components of our proposed method compared with the CNN method (Ke *et al.*, 2018 ▸) on 64 images

	CNN			ORB+MLP			
Processes	LCN	CNN	Total time	ORB	BVWs	MLP	Total time
Train	5700	260	5960	11.264	3171	197.3	3379.564
Test	5700	50	5750	11.584	3099	101.03	3211.614

**Table 6:** Classification performance (%) with cross-data-set training and testing with ORB+MLP Numbers in bold indicate the best performance.

Train/test	LO19	L498	LN83	LN84
LO19	**92.0**	73.3	75.7	77.5
L498	69.7	**89.7**	71.5	67.1
LN83	72.3	78.4	**93.5**	80.1
LN84	79.1	75.9	72.6	**96.5**
